# Thermo-cautery, and Its Use in Veterinary Practice

**Published:** 1898-02

**Authors:** P. O. Koto

**Affiliations:** Forest City, Iowa


### THERMO-CAUTERY, AND ITS USE IN VETERINARY PRACTICE.

By P. O. Koto, M.D.C.,

FOREST CITY, IOWA.

Providence, perhaps, never bestowed on mankind a greater gift than the horse, the noblest beast of burden, and man's best friend; but, with its wonderful anatomical construction and graceful movements, it is oftentimes, like modern machinery, liable to accidents and breakdowns, requiring the aid of an expert. No class, breed, size, or sex is exempt from the various ailments liable to follow from overwork, lack of exercise, idleness, excessive play, as well as abuse by its inhuman master. Interruption with the free movement of any part of the body and limbs causes lameness and untold suffering to the animal, and lack of usefulness and depreciation in value to its owner. Lameness, when severe, is usually easy to locate; but when slight, with no history of the case, requires a critical examination. Mistaken diagnosis often happens with the inexperienced.

The methods of treatment are numerous: Hot and cold applications, compresses, poultices, absorbent and stimulating liniments, blisters, cauterization, and a great many other methods.

There are three kinds of cauterization: superficial, objective, and potential. These may be subdivided.

The actions of ordinary blisters are usually too superficial to exert much influence on deep-seated ailments. The *actual cautery*, on account of the more intense and penetrating inflammation produced, is more effective. The difference between potential and actual cautery is—potential cauterization consists in the application to the surface of some chemical agent (caustic or acid), destroying the skin and tissues. Objective cauterization is now seldom practised; it consists in cauterizing the diseased parts by means of a hot iron held at a certain distance from it. Actual cauterization is not a new invention, but its value has been recognized from remote ages up to the present time; not a barbarous, inhuman method, as claimed by those that are prejudiced. In certain cases it is less painful and far excels other methods of treatment.

In the hands of those unskilled in its legitimate use, the damage done may be equal and even greater than the benefits derived. The operation does not merely consist in burning and searing with a hot iron, producing an extensive sore; it consists in the skilful and mechanical method of introducing heat into the living body with a special object in view. In some cases the object is to form a permanent inelastic and unyielding bandage to the limbs or parts by causing a shrinkage of the skin and tissues. In deep-seated complaints a more active inflammation is produced, causing a greater flow of blood to the parts, assisting nutrition to the existing morbid structure; the more highly organized parts having the most sensibility, hence causing the most pain.

The ordinary instruments employed for actual cauterization are made from iron, steel, and copper. The most modern improvement is the thermo-cautery of various makes, the original being invented by Dr. Paquelin. After the discovery, by the engineer Montaigne, of the incandescent light produced by the combustion of hydrocarbon gas heated in a platinum shell, and used in the mines, Dr. Paquelin introduced it to the medical profession for cauterizing purposes. Many improvements have since been made. There are various makes on the market, nearly all consisting of a reservoir-handle, or plated cylinder, about four or five inches long, packed with absorbent non-combustible fibre, held in position by a metal netting.

Hydrocarbon gas being generated by means of gentle pressure to a double rubber-bulb attached to the reservoir, containing benzine or other hydrocarbons, the continuous stream of air thrown upon the surface of the benzine becomes mechanically charged with benzoline vapor; the mixed gases pass out of the chamber or handle into another tube or shell, containing two perforations for exit of the gases resulting from combustion, a platinum point or hood, of various shapes and sizes, being attached to this. By heating the point with a spirit-lamp or patent blowpipe, attached to the reservoir by means of a rubber tube and stopcock, gentle pressure of the rubber-bulb forces the mixed air or vapor to the heated platinum points, causing a chemical union of the two gases, and complete incandescence is obtained. When properly charged it should keep the points burning from one and one-half to two hours.

The thermo-cautery is in every respect, for most purposes, preferable to the ordinary cautery-irons, being more convenient to handle, extremely light and portable, and can be employed away from home, requiring no forge, there being, consequently, less danger from fire when employed in an ordinary barn. It is very clean and attractive, and will not corrode, scale, or oxidize; hence, no danger from the introduction of foreign bodies. The continuous, even heat in a small space does not roast the skin adjoining the lines, will leave a smaller blemish, and is less painful. By continuous practice it is more easily applied, and, unless the animal is very vicious, or of a nervous disposition, the operation can be performed with the animal in a standing position, a hood being applied to the head, a twitch to the lip, and one limb being held up by a leg-strap, side line, or assistant. If the animal is of a very irritable and determined disposition he should be placed on an operating-table, or be firmly secured by the aid of throwing-harness. Where the operation is very painful, the animal may be placed under the influence of an anæsthetic agent. If both sides of the limb are to be operated on, operate on the inner side first, for if the external side should be cauterized first and turned over on to the parts it would be more liable to injury.

In preparing for the operation the hair should be thoroughly removed by a clipper, or shaved; cauterizing without removing the hair is liable to burn the surface. After removing the hair, brush the parts, or wash thoroughly, and apply some antiseptic, and rub dry before applying the cautery. Never fire a recent injury; for instance, a sprained tendon. First reduce the fever by cold or warm applications; warm applications combined with bandaging will assist reabsorption. For a diseased tendon, apply two long rolls of tow, oakum or cotton, rather thicker than a man's thumb, moistened in warm water and placed lengthwise, the tendon held in place by linen or cotton bandages wetted and applied rather tightly; over this place a dry woollen bandage, and change every four hours, continued for a few days.

There are many forms of cauterizing, but the most ordinary are the penetrant and linear. These lines or points may be arranged in a number of ways, according to situation or fancy of the operator: Parallel lines, diagonal, vertical, rectangular, triangular, and oblique—the more simple the design the better. Lines made in the shape of a lyre are often used, but the most ordinary is the feather-shape. If convenient, run the lines in an oblique direction of the limb, so that the skin, yielding to the movements of the region it covers, will have a tendency to bring the edges together and leave the blemish more completely covered by hair. The distance between the lines will depend upon the depth to be cauterized, also the situation and fineness of the skin. Begin by first marking the outlines with the cautery just hot enough to burn the hair. In certain locations the outlines can be made with crayon, and afterward traced lightly by the cautery. Then heat the points to a bright red or cherry color (avoid a white heat). Do not cauterize too deeply at first, but go over it several times, until the desired color is obtained. These may be divided into three groups: The first is of a golden-yellow, the lines narrow and shallow, with very slight serosity; the second is a bright yellow, with a slight reddish exudation, the lines deeper than the first; the third being a light yellow, with an abundant serous flow.

The veterinarian familiar with the methods of a few incompetent trainers at the race-tracks has often witnessed a speedy animal brought to the track slightly lame and forced to go three to five miles up to speed striking a 700-pound blow or more. Fortunes have been lost and bright prospects ruined by overworking the young animal. Almost every breeder and owner can point to such a case in his own career. Yet accidents will follow, under the most favorable circumstances, by the most able and careful trainers, resulting in strained or ruptured tendons and ligaments, the location of the complaint often depending upon the build and conformation of the animal. Perhaps those most often met with in young animals are the distention of the lower tendinous sheaths of the extensor and flexor tendons. In older animals sprains and ruptures of the suspensory ligament are often met with, either single or double, or in company with inferior check-ligament, in some cases the fetlock-joint almost descending to the ground, which requires special treatment. Lower the toe by paring the hoof, or raise the heel by shoeing, or brace the limbs by means of a truss attached to the shoe and fastened to the limb by straps. Frequently we find sprains and ruptures of the flexor pedis perforatus, perforans tendons, and tendinous sheaths, and check-ligament, and sometimes involving the sesamoidean sheath.

Both the penetrant- and feather-points are employed in the treatment of these tendons and suspensory ligaments. Feather-fire from behind downward and forward, and make the lines from three-quarters to one inch apart, the depth depending upon the severity of the case. When deep-seated, pin-fire at intervals over and into the tendons and ligaments. For the descending fetlock-joint, pin-fire along the suspensory, downward and forward, to the attachment of the extensor pedis. In some chronic cases the thickening refuses to yield, and the tissues remain firmly organized; then cauterize deeply, and combine with a blister. The chief effect produced in cauterizing is the cicatricial shrinkage of the cutis and tissues—acting as a brace or bandage.

In the treatment of any periostosis or exostosis, such as splints, ringbones, and spavins, both points are employed; but the penetrant or puncture in such cases is more effectual than feather-fire.

The main object is to set up a deep artificial inflammation. The benefit derived is in the direct proportion to the periosteal irritation which it excites. For bone-spavin feather-fire as high up as joint of astragalus, and also on external aspect of joint. A complete anchylosis of the large metatarsal bone and those adjoining above may be produced and still have lameness from the external side. Puncture deeply in the lines over the spavin. The point should be quickly removed if the joint has been entered. Batazzi recently recommended the treatment of spavin by subcutaneous firing, formerly introduced by Nanzio. After making an incision one and one-half to two inches in length over the exostosis, the edges of the wound are drawn back and a few punctures made in the shape of a triangle with the base directed upward. No doubt great assistance in the treatment of spavins and ringbones could be attained by the x-ray if it could be practically applied. After cauterizing, blister the parts and place in single stall, and tie head up high to avoid biting the parts. Do not let the animal lie down for four weeks. At the end of six weeks turn loose in a box-stall. In eight or ten weeks give gradual exercise, but the longer the rest the better. Repeat the treatment if necessary. Failure to cure will often follow and baffle the skill of the practical veterinarian by this and all other methods of treatment.

Atrophy of the crural and gluteal muscles is often the sequel of azoturia. Its connection with the disease is explained by the fact that the crural nerve passes through the ilio-psoas muscles, which in this disease seem particularly involved. Puncture deeply over the diseased surface. The benefit obtained is through the deep-seated inflammation produced checking degenerative changes and improving nutrition to the paralyzed muscle. During the season of 1894 a fine young gelding, weighing about 1400 pounds, became my property upon the payment of one dollar. The animal about three months previously suffered from azoturia, and could only walk with great difficulty, the muscle from the ilium to the patella being completely atrophied. The penetrant cautery was employed, and at the end of three months the muscle was completely restored, resulting in a permanent cure, and the horse brought fifty dollars. I can point to a number of similar cases.

There are many other ailments in which treatment with the thermo-cautery is of great value: Atrophy of the anterior and posterior spinatus muscles, curbs, partial luxation of the patella, hydrarthrosis, synovitis, sesamoiditis, arthritis, paraplegia, for the removal of warts and tumors, to evacuate and dilate cavities, fistula, poll-evils, polypus, piles; in the treatment of gangrenous wounds and ulcers, actinomycosis, etc. Its application in surgery is of great benefit, and it should be in the hands of every practising veterinary surgeon.

